# Association between Intrapancreatic Fat Deposition and Lower High-Density Lipoprotein Cholesterol in Individuals with Newly Diagnosed T2DM

**DOI:** 10.1155/2023/6991633

**Published:** 2023-01-28

**Authors:** Jianliang Wang, Qingyun Cai, Xiaojuan Wu, Jiaxuan Wang, Xiaona Chang, Xiaoyu Ding, Jia Liu, Guang Wang

**Affiliations:** ^1^General Surgery Department, Beijing Chao-Yang Hospital, Capital Medical University, Beijing 100020, China; ^2^Department of Endocrinology, Beijing Chao-Yang Hospital, Capital Medical University, Beijing 100020, China

## Abstract

**Background:**

Intrapancreatic fat deposition (IPFD) usually occurs in individuals with type 2 diabetes mellitus (T2DM), but its physiopathological influence remains controversial. The present study aimed to investigate IPFD and its associations with various aspects of glucose and lipid metabolism in individuals with newly diagnosed T2DM.

**Methods:**

A total of 100 individuals were included, consisting of 80 patients with newly diagnosed T2DM and 20 age- and sex-matched healthy controls. Then, we assessed IPFD using magnetic resonance imaging (MRI) and various parameters of glucose and lipid metabolism.

**Results:**

Individuals with newly diagnosed T2DM had a significantly higher IPFD (median: 12.34%; IQR, 9.19–16.60%) compared with healthy controls (median: 6.35%; IQR, 5.12–8.96%) (*p* < 0.001). In individuals with newly diagnosed T2DM, IPFD was significantly associated with FINS and HOMA-IR in unadjusted model (*β* = 0.239, *p*=0.022; *β* = 0.578, *p*=0.007, respectively) and adjusted model for age and sex (*β* = 0.241, *p*=0.022; *β* = 0.535, *p*=0.014, respectively), but these associations vanished after adjustment for age, sex, and BMI. The OR of lower HDL-C for the prevalence of high IPFD was 4.22 (95% CI, 1.41 to 12.69; *p*=0.010) after adjustment for age, sex, BMI, and HbA1c.

**Conclusions:**

Lower HDL-C was an independent predictor for a high degree of IPFD.

## 1. Introduction

The occurrence and progression of T2DM are associated with system fat distribution and local fat deposition, beyond only obesity. Hepatic fat deposition substantially contributes to the development of insulin resistance and inflammatory complications [1]. Ectopic fat depositions in muscle, pericardium, perivascular, and renal sinus were reported to exert adverse effects on metabolism diseases as well [2]. The pancreas is another important metabolic organ, but the physiopathological influence of intrapancreatic fat deposition (IPFD) remains unclear, even though this link was discovered almost a century ago [3]. Recent study suggested that individuals with T2DM showed a significantly higher IPFD compared to healthy individuals [4], which might lead to *β*-cell dysfunction, hyperglycemia, and other associated complications [2, 5].

To date, the use of chemical shift-encoded magnetic resonance imaging (MRI) for IPFD had shown a near-perfect correlation with fat content in the phantoms [6], especially since it had been validated against histology [7]. Therefore, MRI has become the most naturally fit imaging modality for the noninvasive quantification of IPFD in humans [8]. We quantified IPFD by MRI and various aspects of glucose and lipid metabolism and assessed their associations among individuals with newly diagnosed T2DM.

## 2. Methods

### 2.1. Subjects

This study enrolled 80 individuals with newly diagnosed T2DM in the Department of Endocrinology, Beijing Chaoyang Hospital affiliated to Capital Medical University, from November 2018 to October 2019. Additionally, 20 age- and sex-matched healthy individuals who had undergone a routine physical examination were included as the healthy control group at the same hospital. All T2DM individuals must be diagnosed within 3 months, based on the American Diabetes Association (ADA) diagnostic criteria [9]. To avoid the interference of diabetes treatment on IPFD and metabolic parameters, all eligible individuals should not have a history of receiving any diabetes treatment, such as intensive lifestyle interventions or taking hypoglycemic agents. Meanwhile, all individuals should not have any history of smoking and alcohol consumption (>70 g/week for women or >140 g/week for men). Individuals with ongoing or previous use of any medications known to affect lipid metabolism (including other statins, fibric acid derivatives, nicotinic acid, cholestyramine, ezetimibe, or omega-3 fatty acids) were also excluded. All participants had given written informed consent before enrollment. This study was approved by the Ethics Committee of Beijing Chaoyang Hospital affiliated to Capital Medical University.

### 2.2. Anthropometry and Metabolite Assays

All participants underwent a detailed medical history collection and physical examination at enrollment. Their blood specimens were drawn from the antecubital vein after overnight fasting. Fasting blood glucose (FBG) was measured by the glucose oxidase method (Hitachi 747, Roche Diagnostics). Fasting insulin (FINS) was estimated by the chemiluminescence method (Dimension Vista, Siemens Healthcare Diagnostics). Hemoglobin A1c (HbA1c) was measured by high-performance liquid chromatography, using an HLC-723G7 analyzer (Tosoh Corporation, Tokyo, Japan).

The serum levels of total cholesterol (TC), triglyceride (TG), low-density lipoprotein cholesterol (LDL-C), and high-density lipoprotein cholesterol (HDL-C) were measured by colorimetric enzymatic assays using an autoanalyzer (Hitachi 7170). Homeostasis model assessment of insulin resistance (HOMA-IR) and homeostasis model assessment of *β*-cell function (HOMA-*β*) were calculated from the FBG and FINS according to the formula described by Matthews: HOMA-IR = FINS (*µ*IU/mL)*∗*FBG (mmol/L)/22.5 and HOMA-*β* = 20*∗*FINS (*µ*IU/mL)/[FBG (mmol/L) − 3.5] [10].

### 2.3. Intrapancreatic Fat Quantification

MRI examinations were performed with a 3.0-T whole-body human MRI scanner (Magnetom Prisma; Siemens Healthcare, Erlangen, Germany). The scanning protocol comprised an initial set of localizer images and then a T1 volumetric interpolated breath-hold examination (VIBE) Dixon sequence. Regions of the pancreas were placed after the image was acquired, followed by determination of fat content of each voxel. The imaging parameters were set as follows: TE1 of 1.23 ms, TE2 of 2.46 ms, TR of 3.97 ms, 9° flip angle, bandwidth 1 of 1040 Hz/Px, bandwidth 2 of 1040 Hz/Px, and a slice thickness of 3.0 mm. All participants were instructed to fast overnight for 10 to 12 hours before imaging and hold their breath during end expiration to ensure consistency.

The regions of interest (ROIs) covered the entire pancreas, ensuring devoid of large vessels, ducts, organ boundaries, focal pancreatic lesions, and imaging artifacts. Then, Siemens Syngo workstation and MITK 3M3 software were used for imaging processing and quantification. The same radiologist performed both image processing and data readout for each group, and a blinded radiologist with >4-year experience guaranteed the quality of all data and images.

### 2.4. Data Analysis

Data analyses were conducted with SPSS 25.0 (SPSS Inc., Chicago, IL, USA). Normally distributed variables were presented as mean ± standard deviation (SD), and skewed-distribution variables were presented as median and interquartile range (IQR). Data of categorical variables were expressed as number (%).Student's *t*-test, the Mann–Whitney *U* test, or chi-square test was used to investigate the differences in baseline characteristics between T2DM group and healthy control group.

To determine the associations between IPFD and glucose metabolism indicators in individuals with newly diagnosed T2DM, we compared *β* coefficients by regression analyses. The univariate analyses and multiple variable analyses were conducted as unadjusted model (model 1); adjusted model for age and sex (model 2); and adjusted model for age, sex, and BMI (model 3).

To determine the association between IPFD and lipid metabolism parameters, the odds ratios (ORs) and 95% confidence intervals (CIs) were calculated using ordinal logistic regression analysis. The analyses included three models: model 1 was unadjusted; model 2 was adjusted for age and sex; and model 3 was adjusted for age, sex, BMI, and HbA1c. A significance level of *p* value <0.05 was adopted.

## 3. Results

### 3.1. Characteristics of Study Participants

**Figure 1:**
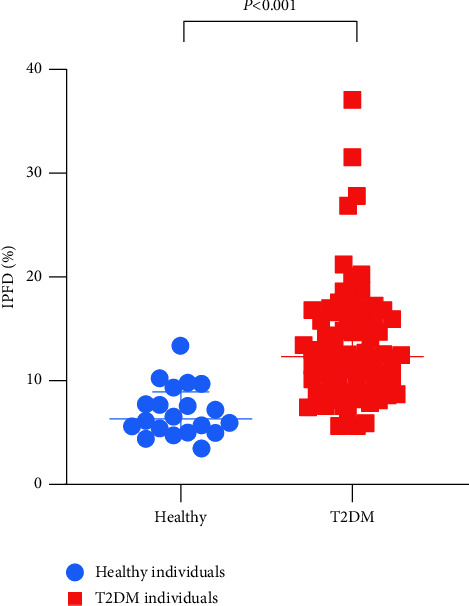
IPFD (median and IQR) in the healthy individuals and individuals with newly diagnosed T2DM (*p* < 0.001 by Mann–Whitney *U* test).

As summarized in Table 1, 80 individuals with newly diagnosed T2DM were recruited into this study, comprising 60 men and 20 women with a mean age of 42.89 ± 10.93 years. 20 age- and sex-matched healthy individuals were also included. The mean BMI (kg/m^2^) in the newly diagnosed T2DM and healthy individuals was 29.74 ± 4.81 and 21.37 ± 1.85, respectively. Individuals with newly diagnosed T2DM had a significantly higher IPFD with a median (IQR) of 12.34 (9.19–16.60) % compared with 6.35 (5.12–8.96) % in the healthy individuals (Figure 1). Difference in glucose and lipid profiles was observed between the two groups. Individuals with newly diagnosed T2DM had higher FBG, FINS, HbA1c, HOMA-IR, TC, and TG as well as lower HOMA-*β* and HDL-C than the healthy individuals (*p* < 0.05) (Table 1).

### 3.2. Associations between IPFD and Glycemic Parameters in Individuals with Newly Diagnosed T2DM

The median (IQR) level of FINS was 10.85 (8.25–15.25) *μ*U/mL. IPFD was significantly associated with FINS in model 1 (*β* = 0.239; 95% CI, 0.036 to 0.442; *p*=0.022) and model 2 (*β* = 0.241; 95% CI, 0.037 to 0.445; *p*=0.022) (Table 2).

The median (IQR) level of HOMA-IR was 4.83 (3.74–6.77). IPFD was significantly associated with HOMA-IR in model 1 (*β* = 0.578; 95% CI, 0.165 to 0.990; *p*=0.007) and model 2 (*β* = 0.535; 95% CI, 0.110 to 0.960; *p*=0.014) (Table 2).

The median (IQR) levels of FBG, HbA1c, and HOMA-*β* were 9.98 (8.25–12.40) mmol/L, 10.30 (8.90–11.33) mmol/mol, and 34.05 (21.64–59.20) %, respectively. There was no significant association between IPFD and all these parameters in both unadjusted and adjusted models (Table 2).

### 3.3. Associations between High IPFD and Dyslipidemia in Individuals with Newly Diagnosed T2DM

Due to the lack of definition of high IPFD in T2DM individuals, we consulted the upper tertiles (14.84%) in this study as the diagnostic threshold. According to the 2016 Chinese guideline for the management of dyslipidemia in adults (2018), the criteria of dyslipidemia are as follows: (1) TC ≥ 5.2 mmol/L; (2) TG ≥ 1.7 mmol/L; (3) LDL-C ≥ 2.6 mmol/L; and (4) HDL-C < 1.0 mmol/L [11].

Among 80 patients with newly diagnosed T2DM, 47 patients had lower HDL (<1.0 mmol/L). The median (IQR) level of HDL-C was 0.90 (0.80–1.10) mmol/L. The OR of low HDL-C (<1.0 mmol/L) for the prevalence of high IPFD (≥14.84%) was 3.67 (95% CI, 1.43 to 9.44; *p*=0.007) in model 1; 4.34 (95% CI, 1.52 to 12.39; *p*=0.006) in model 2; and 4.22 (95% CI, 1.41 to 12.69; *p*=0.010) in model 3 (Table 3).

The median (IQR)/mean ± SD levels of TC, TG, and LDL-C were 5.43 (4.61–6.02) mmol/L, 2.22 (1.57–4.05) mmol/L, and 3.10 ± 1.08 mmol/L, respectively. In any model, none of the three adverse lipid status were associated with high IPFD (Table 3).

## 4. Discussion

The results of this cross-sectional study showed that individuals with newly diagnosed T2DM exhibited higher BMI and IPFD levels as well as an impaired state of glucose and lipid metabolism compared with healthy individuals. IPFD was significantly associated with FINS and HOMA-IR, and these associations vanished after adjustment for age, sex, and BMI. Interestingly, we further confirmed that lower HDL-C was independently associated with a high degree of IPFD, rather than high TC, TG, and LDL-C.

Although it is widely accepted that T2DM commonly coexists with IPFD [12], the physiopathological influence of IPFD remains controversial. Some studies found that IPFD was significantly associated with insulin resistance and *β*-cell function [13–15]; however, others hold a negative view [6, 16–18]. A recent study demonstrated that lipid droplet accumulation in *β* cells was associated with insulin resistance in T2DM individuals [19]. Ectopic fat deposition, including hepatic and pancreatic fat, was actuated by adipocyte dysfunction and elevated free fatty acids (FFAs) which were derived from triglyceride degradation, all of which might be involved in the process of insulin resistance [20]. In this study, we observed that IPFD was significantly associated with FINS and HOMA-IR. However, this association vanished after further adjustment for BMI.

In addition, we also found that IPFD was not statistically associated with *β*-cell function in individuals with newly diagnosed T2DM. This irrelevance could be partly explained by methodological (such as insufficient enrollment and inevitable limitation of the HOMA model) and pathophysiological factors. Firstly, the pancreatic *β* cells had a strong compensatory capacity to tolerate the adverse effects of ectopic fat deposition [21], and individuals with newly diagnosed T2DM might remain a certain extent of this adapted ability. Secondly, the simultaneous activation of several deleterious cascades might further accelerate *β*-cell dysfunction, including oxidative stress, chronic inflammation, hypoperfusion of islets, and *β*-cell apoptosis, and thus the deterioration of *β*-cell function developed at a rate disproportionate to pancreatic fat deposition [22].

Although it was frequently observed that obese individuals had IPFD, the development of IPFD might not always be a consequence of obesity [11]. The associations between IPFD and dyslipidemia had been investigated in obese individuals and individuals with a history of acute pancreatitis [12, 23]; however, we focused on individuals with newly diagnosed T2DM. Notably, this study showed that lower HDL-C, rather than other types of dyslipidemia, was independently associated with IPFD. On the one hand, one of the key factors in reverse cholesterol transport, HDL-C, mediated the clearance of peripheral lipids and therefore contributed to the reduction of ectopic fat deposition. On the other hand, the anti-inflammatory properties of HDL-C might also inhibit the chronic inflammatory response of ectopic deposited adipocytes.

This study had several limitations. Firstly, this cross-sectional study cannot evaluate the risk for glucose and lipid metabolism associated with IPFD and draw causal inferences. Secondly, due to the lack of relevant data for the OGTT, we were unable to calculate the disposition index and further assess the *β*-cell function in the context of insulin resistance. Thirdly, due to the inhomogeneous distribution and different mechanisms of ectopic fat in the various pancreatic compartments [24, 25], this MRI study performed a volumetric evaluation of IPFD, which failed to accurately determine the complex pathological influence. Lastly, information on the history of physical activity of participants was lacking, which might affect HDL-C levels. Further research studies are needed to consider the interference of these factors.

In conclusion, IPFD was significantly associated with defective glucose and lipid metabolic status in individuals with newly diagnosed T2DM. IPFD was associated with FINS and HOMA-IR, but these associations vanished after further adjustment for BMI, which indicated that IPFD might be a potential accomplice in the progression of insulin resistance. Interestingly, we further found that lower HDL-C was an independent predictor for a high degree of IPFD in individuals with newly diagnosed T2DM. In the future, more multifaceted research is suggested to gain much insight into the contributing role of HDL-C to the progress of IPFD.

## Figures and Tables

**Table 1:** Characteristics of study individuals.

Parameters	Healthy controls (*n* = 20)	T2DM (*n* = 80)	*P*
Male sex, *n* (%)	13 (65)	60 (75)	0.404
Age (years)	42.55 ± 13.14	42.89 ± 10.93	0.753
BMI (kg/m^2^)	21.37 ± 1.85	29.74 ± 4.81	<0.001^*∗∗∗*^
IPFD (%)	6.35 (5.12–8.96)	12.34 (9.19–16.60)	<0.001^*∗∗∗*^
FBG (mmol/L)	4.69 (4.46–4.85)	9.98 (8.25–12.40)	<0.001^*∗∗∗*^
FINS (*μ*U/mL)	5.75 (4.60–6.48)	10.85 (8.25–15.25)	<0.001^*∗∗∗*^
HbA1c (mmol/mol)	5.15 (5.10–5.30)	10.30 (8.90–11.33)	<0.001^*∗∗∗*^
HOMA-IR	1.22 (0.94–1.42)	4.83(3.74–6.77)	<0.001^*∗∗∗*^
HOMA-*β* (%)	89.26 (71.26–115.70)	34.05 (21.64–59.20)	<0.001^*∗∗∗*^
TC (mmol/L)	4.70 (4.26–4.99)	5.43 (4.61–6.02)	<0.001^*∗∗∗*^
TG (mmol/L)	0.94 (0.64–1.55)	2.22 (1.57–4.05)	<0.001^*∗∗∗*^
LDL-C (mmol/L)	2.73 ± 0.61	3.10 ± 1.08	0.050
HDL-C (mmol/L)	1.29 (1.08–1.66)	0.90 (0.80–1.10)	<0.001^*∗∗∗*^

Data are expressed as mean ± SD, median (interquartile range), or *n* (%). BMI, body mass index; FBG, fasting blood glucose; FINS, fasting insulin; HbA1c, hemoglobin A1c; HOMA-IR, homeostasis model assessment of insulin resistance; HOMA-*β*, homeostasis model assessment of *β*-cell function; TC, total cholesterol; TG, triglyceride; LDL-C, low-density lipoprotein cholesterol; HDL-C, high-density lipoprotein cholesterol. ^*∗∗∗*^*p*  <  0.001. The significance of bold values indicates that the differences of these parameters between the two groups were statistically significant (*P* value < 0.05).

**Table 2:** Associations between IFPD and glycemic parameters in 80 individuals with newly diagnosed T2DM.

Parameters	Model 1		Model 2		Model 3	
	*β* (95% CI)	*P*	B (95% CI)	*P*	B (95% CI)	*P*
FBG	0.204 (−0.235, 0.643)	0.358	0.082 (−0.368, 0.533)	0.717	−0.050 (−0.471, 0.371)	0.812
FINS	0.239 (0.036, 0.442)	**0.022** ^*∗*^	0.241(0.037, 0.445)	**0.022** ^*∗*^	0.127 (−0.078, 0.333)	0.221
HbA1c	−0.046(-0.637, 0.545)	0.878	−0.169 (−0.761, 0.422)	0.570	−0.068 (−0.616, 0.481)	0.807
HOMA-IR	0.578 (0.165, 0.990)	**0.007** ^*∗∗*^	0.535 (0.110, 0.960)	**0.014** ^*∗*^	0.263 (−0.178, 0.704)	0.238
HOMA-*β*	0.015 (−0.028, 0.059)	0.488	0.021 (−0.023, 0.064)	0.351	0.009 (−0.032, 0.050)	0.663

Regression analysis was used to determine the associations between IPFD and glucose metabolism indicators in individuals with newly diagnosed T2DM. Model 1: unadjusted; model 2: adjustment for age and sex; model 3: adjustment for age, sex, and BMI. FBG, fasting blood glucose; FINS, fasting insulin; HbA1c, hemoglobin A1c; HOMA-IR, homeostasis model assessment of insulin resistance; HOMA-*β*, homeostasis model assessment of *β*-cell function. ^*∗*^*p*  <  0.05; ^*∗∗*^*p*  <  0.01.

**Table 3:** Associations between high IPFD and dyslipidemia in 80 individuals with newly diagnosed T2DM.

Variables	Model 1		Model 2		Model 3	
	OR (95% CI)	*P*	OR (95% CI)	*P*	OR (95% CI)	*P*
Higher TC	0.88 (0.38–2.04)	0.773	0.88 (0.37–2.07)	0.767	0.91 (0.38–2.21)	0.843
Higher TG	2.61 (1.07–6.41)	0.036^*∗*^	2.16 (0.83–5.57)	0.113	2.07 (0.78–5.48)	0.142
Higher LDL-C	1.13 (0.50–2.57)	0.773	1.07 (0.46–2.49)	0.884	0.95 (0.40–2.28)	0.911
Lower HDL-C	3.67 (1.43–9.44)	0.007^*∗∗*^	4.34 (1.52–12.39)	0.006^*∗∗*^	4.22 (1.41–12.69)	0.010^*∗*^

Ordinal logistic regression analysis was used to determine the associations between IPFD and adverse lipid metabolic status in individuals with newly diagnosed T2DM. Model 1: unadjusted; model 2: adjustment for age and sex; model 3: adjustment for age, sex, BMI, and HbA1c. TC, total cholesterol; TG, triglyceride; LDL-C, low-density lipoprotein cholesterol; HDL-C, high-density lipoprotein cholesterol. ^*∗*^*p*  <  0.05; ^*∗∗*^*p*  <  0.01. The significance of bold values indicates that the associations between IPFD and adverse lipid metabolic status in particular models were statistically significant (*P* value < 0.05).

## Data Availability

The data used to support the findings of this research are available on request from the corresponding author.

## Abbreviations

T2DM: Type 2 diabetes mellitus
IPFD: Intrapancreatic fat deposition
MRI: Magnetic resonance imaging
BMI: Body mass index
FBG: Fasting blood glucose
FINS: Fasting insulin
HbA1c: Hemoglobin A1c
HOMA-IR: Homeostasis model assessment of insulin resistance
HOMA-*β*: Homeostasis model assessment of *β*-cell function
TC: Total cholesterol
TG: Triglyceride
LDL-C: Low-density lipoprotein cholesterol
HDL-C: High-density lipoprotein cholesterol
FFAs: Free fatty acids
SD: Standard deviation
IQR: Interquartile range
ROIs: Regions of interest
OR: Odds ratio
CI: Confidence interval.
